# Supplementary material for: ProteinShader: illustrative rendering of macromolecules
Source: BMC Struct Biol. 2009 Mar 30;9:19. doi: 10.1186/1472-6807-9-19 (PMC2672931; doi:10.1186/1472-6807-9-19)
Supplement: Additional file 1 — ProteinShader program without source code. This compressed file contains the complete ProteinShader program including associated libraries, but no source code. A README.txt file gives an overview of the ProteinShader distribution, and the index.html file in the help subdirectory has directions on getting started with the program as well as a set of tutorials. [file 1472-6807-9-19-S1.zip › ProteinShader-beta-0_9_4-binary/help/api/org/proteinshader/gui/listeners/controlpanel/OptimizePanelListenerFactory.html]

OptimizePanelListenerFactory (ProteinShader API)


|  |  |  |
| --- | --- | --- |
| **Overview** \| **Package** \| **Class** \| **Use** \| **Tree** \| **Deprecated** \| **Index** \| **Help** | |  |
| **PREV CLASS**   **NEXT CLASS** | **FRAMES**    **NO FRAMES**     **All Classes** |  |
| SUMMARY: NESTED \| FIELD \| CONSTR \| METHOD | DETAIL: FIELD \| CONSTR \| METHOD |  |


---


## org.proteinshader.gui.listeners.controlpanel Class OptimizePanelListenerFactory

```
java.lang.Object
  org.proteinshader.gui.listeners.controlpanel.OptimizePanelListenerFactory
```

---

``` public class OptimizePanelListenerFactory extends Object ```

Creates the listener for the OptimizePanel.

---

**Constructor Summary**

`OptimizePanelListenerFactory(Mediator mediator,
OptimizePanel optimizePanel)`
  
          Constructs a OptimizePanelListenerFactory.


| **Method Summary** | |
| --- | --- |
| `ActionListener` | `createApplyButtonActionListener()`             Creates an action listener for the "Apply" button. |
| `ItemListener` | `createAutoTilingButtonItemListener()`             Creates a change listener for the "Auto Tiling" check box button. |
| `ActionListener` | `createCylinderCapsButtonActionListener()`             Creates an action listener for the "Cylinder Caps" radio button. |
| `ActionListener` | `createCylinderCapsMenuActionListener()`             Creates an action listener for the "Cylinder Caps" menu. |
| `ActionListener` | `createCylindersButtonActionListener()`             Creates an action listener for the "Cylinders" radio button. |
| `ActionListener` | `createCylindersMenuActionListener()`             Creates an action listener for the "Cylinders" menu. |
| `ActionListener` | `createDefaultButtonActionListener()`             Creates an action listener for the "Default" button. |
| `ActionListener` | `createSpheresButtonActionListener()`             Creates an action listener for the "Spheres" radio button. |
| `ActionListener` | `createSpheresMenuActionListener()`             Creates an action listener for the "Spheres" menu. |
| `ChangeListener` | `createSpinnerChangeListener()`             Creates a change listener for a spinner. |

**Methods inherited from class java.lang.Object**

`clone, equals, finalize, getClass, hashCode, notify, notifyAll, toString, wait, wait, wait`

**Constructor Detail**

### OptimizePanelListenerFactory

```
public OptimizePanelListenerFactory(Mediator mediator,
                                    OptimizePanel optimizePanel)
```

:   Constructs a OptimizePanelListenerFactory.

    **Parameters:**: `mediator` - the centralized Mediator object that listeners call on to accomplish their task.: `optimizePanel` - the OptimizePanel that this class creates listeners for.


**Method Detail**

### createAutoTilingButtonItemListener

```
public ItemListener createAutoTilingButtonItemListener()
```

:   Creates a change listener for the "Auto Tiling" check box button.
    This change listener is used to shut automatic tiling (level of
    detail) on/off. When automatic tiling is on, all of the other
    controls in the OptimizePanel will be disabled (grayed out).

    :   **Returns:**: The item listener.

---


### createSpinnerChangeListener

```
public ChangeListener createSpinnerChangeListener()
```

:   Creates a change listener for a spinner. This change listener
    calls on the applySpinnerTilingNumber() method of the
    OptimizePanel.

    :   **Returns:**: The change listener.

---


### createApplyButtonActionListener

```
public ActionListener createApplyButtonActionListener()
```

:   Creates an action listener for the "Apply" button. This action
    listener calls on the applySpinnerTilingNumber() method of the
    OptimizePanel.

    :   **Returns:**: The action listener.

---


### createDefaultButtonActionListener

```
public ActionListener createDefaultButtonActionListener()
```

:   Creates an action listener for the "Default" button. This action
    listener calls on the applyDefaultTilingNumber() method of the
    OptimizePanel.

    :   **Returns:**: The action listener.

---


### createSpheresMenuActionListener

```
public ActionListener createSpheresMenuActionListener()
```

:   Creates an action listener for the "Spheres" menu. This action
    listener calls on the updateSpheresLabel() method of the
    OptimizePanel in order to update the tiling number based on the
    current menu choice. The "Spheres" radio button will also be set
    as selected.

    :   **Returns:**: The action listener.

---


### createCylindersMenuActionListener

```
public ActionListener createCylindersMenuActionListener()
```

:   Creates an action listener for the "Cylinders" menu. This action
    listener calls on the updateCylindersLabels() method of the
    OptimizePanel in order to update the tiling number based on the
    current menu choice. The "Cylinders" radio button will also be
    set as selected.

    :   **Returns:**: The action listener.

---


### createCylinderCapsMenuActionListener

```
public ActionListener createCylinderCapsMenuActionListener()
```

:   Creates an action listener for the "Cylinder Caps" menu. This
    action listener calls on the updateCylinderCapsLabels() method
    of the OptimizePanel in order to update the tiling number based
    on the current menu choice. The "Cylinder Caps" radio button will
    also be set as selected.

    :   **Returns:**: The action listener.

---


### createSpheresButtonActionListener

```
public ActionListener createSpheresButtonActionListener()
```

:   Creates an action listener for the "Spheres" radio button. This
    action listener calls on the setSpinnerToSphereValue() method in
    the OptimizePanel.

    :   **Returns:**: The action listener.

---


### createCylindersButtonActionListener

```
public ActionListener createCylindersButtonActionListener()
```

:   Creates an action listener for the "Cylinders" radio button.
    This action listener calls on the setSpinnerToCylinderValue()
    method in the OptimizePanel.

    :   **Returns:**: The action listener.

---


### createCylinderCapsButtonActionListener

```
public ActionListener createCylinderCapsButtonActionListener()
```

:   Creates an action listener for the "Cylinder Caps" radio button.
    This action listener calls on the setSpinnerToCylinderCapValue()
    method in the OptimizePanel.

    :   **Returns:**: The action listener.


---


|  |  |  |
| --- | --- | --- |
| **Overview** \| **Package** \| **Class** \| **Use** \| **Tree** \| **Deprecated** \| **Index** \| **Help** | |  |
| **PREV CLASS**   **NEXT CLASS** | **FRAMES**    **NO FRAMES**     **All Classes** |  |
| SUMMARY: NESTED \| FIELD \| CONSTR \| METHOD | DETAIL: FIELD \| CONSTR \| METHOD |  |


---

# *Copyright © 2007-2008*
